# The association of metabolic syndrome and Chlamydia pneumoniae, Helicobacter pylori, cytomegalovirus, and herpes simplex virus type 1: The Persian Gulf Healthy Heart Study

**DOI:** 10.1186/1475-2840-5-25

**Published:** 2006-12-01

**Authors:** Iraj Nabipour, Katayon Vahdat, Seyed Mojtaba Jafari, Raha Pazoki, Zahra Sanjdideh

**Affiliations:** 1 Department of Internal Medicine, School of Medicine, Bushehr University of Medical Science, Bushehr, I.R. Iran; 2 Department of Infectious Diseases, School of Medicine, Bushehr University of Medical Science, Bushehr, I.R. Iran; 3 Professor Haghighi Section of Tropical Medicine, The Persian Gulf Health Research Center, Bushehr University of Medical Science, Bushehr, I.R. Iran

## Abstract

**Background:**

The metabolic syndrome together with insulin resistance and their consequences are basic factors in pathogenesis of atherosclerosis. Chronic infections with herpes simplex virus type 1 (HSV-1), cytomegalovirus (CMV), and Chlamydia pneumoniae are associated with the development of atherosclerosis and coronary heart disease. The infectious aspects of metabolic syndrome have not been investigated.

**Methods:**

In a cross-sectional, population-based study, we used National Cholesterol Education Program (NCEP)-Adult Treatment Panel (ATP)-III criteria in 1791 subjects, aged 25 years and over, selected by cluster random sampling in three Iranian ports in the northern Persian Gulf. Sera were analyzed for IgG antibodies to Chlamydia pneumoniae, HSV-1, Helicobacter pylori (H. pylori) and CMV using ELISA.

**Results:**

In multiple logistic regression analysis, of the infectious agents, CMV [OR = 1.81 (1.05–3.10); p = 0.03], H. pylori [OR = 1.50 (1.12–2.00); p = 0.007] and Chlamydia pneumoniae [OR = 1.69 (1.27–2.25); p < 0.0001] showed a significant association with the metabolic syndrome in men and HSV-1 [OR = 1.95 (1.22–3.11); p = 0.005], H. pylori [OR = 1.45 (1.09–1.94); 0.01] and Chlamydia pneumoniae [OR = 1.65 (1.23–2.21); p = 0.001] in women.

**Conclusion:**

The metabolic syndrome, which occurs very frequently in the general population, has a significant association with prior infection with Chlamydia pneumoniae, Helicobacter pylori, cytomegalovirus and herpes simplex virus type 1. Hypothesis about participation of infection in pathogenesis of metabolic syndrome should be investigated.

## Background

The metabolic syndrome is said to consist of a cluster of heart disease risk factors, including low high density lipoprotein cholesterol, high triglycerides, impaired carbohydrate metabolism, central obesity, and high blood pressure [1,2]. The metabolic syndrome is expected to be diagnosed in millions of subjects in the near future worldwide by either WHO or NCEP-ATPIII criteria [3]. Coronary heart disease (CHD), cardiovascular disease (CVD), and total mortality are significantly higher in adults with than in those without metabolic syndrome [4]. The metabolic syndrome is a pro-inflammatory state as evidenced by increased levels of IL-6, TNF-α, and C-reactive protein (CRP), serum amyloid A, and leptin and lower level of adiponectin. Each of the features of the metabolic syndrome in effect contributes to this pro-inflammatory state [5].

Animal/experimental, pathological, and cross-sectional seroepidemiological studies conducted among middle-aged populations provide some support for the hypothesis that infections with herpes simplex virus type 1 (HSV-1), cytomegalovirus (CMV), and Chlamydia pneumoniae are associated with the development of atherosclerosis and coronary heart disease [6].

Infectious agents can contribute to the acceleration of atherosclerosis development by nonspecific mechanisms, such as hypercoagulation, increased production of adhesion molecules, and elevated C-reactive protein (CRP) levels [7].

Given both the high prevalence of IgG antibodies to herpes simplex virus type 1 (HSV-1), cytomegalovirus (CMV), and Chlamydia pneumoniae and the high prevalence of metabolic syndrome among adults, it is particularly important to determine whether serological evidence of prior infection with these agents is associated with metabolic syndrome. We examined this question in an ancillary study to the Persian Gulf Healthy Heart Study, a cohort study of men and women aged ≥ 25 years.

## Materials and methods

The Persian Gulf Healthy Heart Study is a study to determine the risk factors for cardiovascular diseases among the Northern Persian Gulf population (Bushehr and Hormozghan Provinces) and to develop community-based interventional projects to change the lifestyles of the population and to present the rising threat of CVD in the region. The design of this study encompasses two major components: phase I is a cross-sectional prevalence study of unhealthy lifestyle and ischemic heart disease (IHD) and associated risk factors and phase II is a multiple interventional project for reduction of CVD in the region.

### Community sampling and baseline examinations

In phase I of the study, a multiple-stage stratified cluster random sampling technique was used to select 3000 people aged >= 25 years from major ports of Bushehr Province (an Iranian province with the greatest boarder with the Persian Gulf). The studied ports of the Northern Persian Gulf were Bushehr Port (the center of Bushehr Province, with a population of 150000 and coronary events of 481.05 and 156.61 per 100,000 for men and women; respectively), Genaveh and Deilam Ports. Specifications dictated that approximately two persons per selected household could be included in phase I cross-sectional survey.

Examinations were conducted in 2003–04. All subjects were asked to fast and to present to the survey center between 7.30–9.30 a.m. Blood pressure was assessed twice at the right arm after a 15-min rest in the sitting position, using a standard mercury sphygmomanometer. Waist circumference was defined at the midway level between the costal margins and the iliac crests. Hip circumference was measured at the level of the greater trochanters.

A fasting blood sample was taken, all samples were promptly centrifuged, separated and analyses were carried out at the Persian Gulf Health Research Center on the day of blood collection using a Selectra 2 autoanalyzer (Vital Scientific, Spankeren, The Netherlands). Glucose was assayed by enzymatic (glucose oxidase) colorimetric method using a commercial kit (Pars Azmun Inc; Tehran, Iran). Serum total cholesterol and HDL-cholesterol were measured using a cholesterol oxidase phenol aminoantipyrine and triglycerides using a glycerol-3 phosphate oxidase phenol aminoantipyrine enzymatic method. Serum LDL-cholesterol was calculated using the Friedwald formula; LDL-cholesterol was not calculated when triglycerides concentration was >400 mg/dl.

The metabolic syndrome was diagnosed with the criteria indicated by the NCEP-ATP III [8]. According to these criteria, subjects with the metabolic syndrome are those with any combination of three or more of the following risk determinants: fasting plasma glucose >= 6.1 mmol/l, blood pressure >= 130/>= 85 mmHg or antihypertensive treatment, plasma triglycerides >= 1.7 mmol/l, plasma HDL cholesterol < 1.03 mmol/l in men and < 1.29 mmol/l in women, and waist circumference >102 cm in men or >88 cm in women.

### Serology

IgG antibodies against Chlamydia pneumoniae were measured by a commercial test kit (DRG Instruments GmbH, Germany). The principle of the kit was based on an indirect solid-phase enzyme immunoassay with horseradish peroxidase as a marker enzyme; the positivity threshold was enzyme immunounits (EIU) >45. Sera were screened for IgG antibodies against herpes simplex virus type 1, cytomegalovirus and Helicobacter pylori with an ELISA (RADIM SpA, Italia), and the samples were considered positive with IgG values higher than 30 RU/ml for CMV and H. pylori. Samples with optical density higher than cut-off control were considered reactive for anti-HSV type 1 IgG antibodies.

### Statistical methods

For analysis of data, the studied population was divided into four groups: 25–34, 35–44, 45–54, and 55–64 years of age. Odds ratios (ORs) estimating the association of metabolic syndrome with presence of IgG antibodies against infectious agents were calculated.

Multiple logistic regression analysis was used to ascertain the associations between metabolic syndrome and presence of IgG antibodies against infectious agents when the presence of IgG antibodies against infectious agents considered as independent covariates and simultaneously included into the same equation, with metabolic syndrome as the dependent variable. P < 0.05 was considered statistically significant.

Statistical analysis was performed with an IBM computer using the SPSS 9.05 statistical software package (SPSS Inc., Chicago, IL).

## Results

A total of 1791 (49.2% males, 50.8% females) of the studied population were evaluated for IgG HSV-1, CMV, H. pylori and Chlamydia pneumoniae. Of the studied subjects, 36.1% was between 25–34 years, 29.0% between 35–44 years, 21.9% between 45–54 years, and 12.7% between 55–66 years.

A total of 52.1% of the subjects (54.6% of males & 49.9% of females; p = 0.005) had clinical traits of the metabolic syndrome as defined by ATP III criteria. The prevalence of metabolic syndrome was increased with increasing in age (p = 0.0001).

Table 1 shows prevalence of IgG antibodies against HSV-1, Chlamydia pneumoniae, HSV-1 and H. pylori among men & women with and without the metabolic syndrome.

**Table 1:** Unadjusted odd ratios (95% CI) and the prevalence of IgG antibodies against HSV-1, Chlamydia pneumoniae, HSV-1 and H. pylori among men & women with and without the metabolic syndrome; the Persian Gulf Healthy Heart Study

	Male					Female				
	**Metabolic Syndrome**	**Healthy**	O.R*	**C.I†**	**p value**	**Metabolic Syndrome**	**Healthy**	**O.R**	**C.I**	**p value**

**HSV-1‡**	84.2%	83.3%	1.06	0.73–1.54	N.S	92.1%	85.3%	2.01	1.27–3.16	0.003
**CMV§**	94.3%	89.2%	1.99	1.121–3.27	0.007	94.8%	93.6%	1.23	0.7–2.15	N.S
**Chlamydia pneumoniae**	51.9%	37.8%	1.77	1.34–2.32	0.0001	42.2%	30.0%	1.69	1.28–2.23	0.0001
H. pylori**	67.9%	56.6%	1.62	1.23–2.13	0.001	64.4%	55.1%	1.47	1.12–1.92	0.004

* *Odds Ratio*

***† ****Confidence Interval*

***‡ ****Herpes simplex virus type 1*

§ Cytomegalovirus

**Helicobacter pylori

The prevalence of IgG antibodies against Chlamydia pneumoniae was higher in men (45.7%) than women (35.8%) (p < 0.0001); the prevalence of IgG antibodies against HSV-1 was lower in men (83.8%) than women (88.6%) but no significant differences was observed in prevalence of IgG antibodies against CMV and H. pylori in men (92.0%, 62.9%, respectively) and women (94.2% and 59.6%, respectively). There was an increase in IgG antibodies against herpes simplex virus type 1 (HSV-1), H. pylori and Chlamydia pneumoniae with increasing age in both sexes (p = 0.0001) but the increase in prevalence of IgG antibodies against CMV with increasing in age was observed only in men.

The prevalence of IgG antibodies against CMV (p = 0.008), Chlamydia pneumoniae (p < 0.0001), and H. pylori (p < 0.0001) were higher in men with the metabolic syndrome than healthy subjects.

The prevalence of IgG antibodies against HSV-1 (p = 0.003), Chlamydia pneumoniae (p < 0.0001), and H. pylori (p = 0.005) were higher in women with the metabolic syndrome than healthy subjects.

Table 1 shows unadjusted odds ratio (95% C.I) between metabolic syndrome and four infectious agents.

In multiple logistic regression analysis, of the infectious agents, CMV [OR = 1.81 (1.05–3.10); p = 0.03], H. pylori [OR = 1.50 (1.12–2.00); p = 0.007] and Chlamydia pneumoniae [OR = 1.69 (1.27–2.25); p < 0.0001] showed a significant association with the metabolic syndrome in men and HSV-1 [OR = 1.95 (1.22–3.11); p = 0.005], H. pylori [OR = 1.45 (1.09–1.94); 0.01] and Chlamydia pneumoniae [OR = 1.65 (1.23–2.21); p = 0.001] in women.

## Discussion

The metabolic syndrome, which occurs very frequently in our population, had a significant association with chronic viral and bacterial infectious agents. To our knowledge, this is the first population-based report of an association of seropositivity of infectious agents with the metabolic syndrome. The common features of these chronic infectious agents are their contribution to inflammation and promotion of atherosclerosis [6].

### Infection, inflammation and the metabolic syndrome

Endothelial dysfunction was identified both in the experiment and in patients after herpes virus simplex 1 infection, or Helicobacter pylori infection. However, it is not clear whether it is always caused by direct specific activity of a given pathogen or whether it is a result of inflammatory cytokines activity, heat shock protein activity, or CRP activity [9,10]. Individuals infected with multiple pathogens such as HSV-1, HSV-2, CMV and H. pylori have high C-reactive protein levels (markers of inflammation) and the greatest relative risk for coronary artery disease [11]. Thus, pathogens might contribute to the atherosclerotic process by promoting inflammatory response.

All of the characteristics of metabolic syndrome are also modestly associated with elevated levels of CRP. Moreover, CRP levels correlate with other components of the metabolic syndrome that are not easily measured in clinical practice, including fasting insulin, microalbuminuria, and impaired fibrinolysis [12]. A recent study evaluated interrelationships between CRP, the metabolic syndrome, and incident cardiovascular events among 14719 apparently healthy women, 24% of whom had the metabolic syndrome, who were followed-up for an 8-year period for myocardial infarction, stroke, coronary revascularization, or cardiovascular death [13]. Although CRP levels are related strongly to insulin resistance, it is difficult to conclude if low-grade inflammation induces insulin resistance and the metabolic syndrome or is a consequence. A reasonable proposal is that an imbalance in favor of pro-inflammatory cytokines from adipose tissue and other sources triggers CRP secretion. This in turn can exacerbate mild insulin resistance and result in accentuation of other metabolic abnormalities that constitute the metabolic syndrome [5].

Chronic subclinical inflammation is increasingly recognized as a part of the insulin resistance syndrome [14,15]. Dysregulation of the inflammatory axis predicts the development of insulin resistance and type 2 diabetes mellitus. Insulin resistance and another inflammatory state, atherosclerosis, share similar pathophysiological mechanisms, mainly due to the actions of the two major proinflammatory cytokines, TNF-alpha and IL-6 [16].

Taking into account the alleged common inflammatory venue of these conditions, Fisman et al [17] hypothesized that the effects of the interleukins on both disorders would probably moving in the same direction-regardless if harmful, favorable or neutral.

### Proinflammatory cytokines and insulin resistance

TNF-alpha-induced insulin resistance has received much recent attention [18]. Even though predominantly an inflammatory cytokine, it has been implicated in conferring insulin resistance in peripheral tissues in a number of different disease states associated with elevated systemic TNF-alpha levels, such as obesity, cancer, and infection [19]. Chronic exposure of adipocytes to low concentrations of TNF-alpha strongly inhibits insulin-stimulated glucose uptake. Concurrently, TNF-alpha treatment causes a moderate decrease in the insulin-stimulated autophosphorylation of the insulin receptor (IR) and a dramatic decrease in the phosporylation of IR substrate 1, the major substate of the IR in vivo [20]. Thus, TNF-alpha directly interferes with the signaling of insulin through its receptor and consequently blocks biological actions of insulin.

A tight correlation between IL-6 and CRP exists; such a correlation dose not exists for other cytokines. Two important acute phase proteins, CRP and fibrinogen, have IL-6 response elements in the promoter regions of their genes. IL-6 is believed to be the main driver of CRP release from hepatocytes [21]. IL-6 plasma levels are significantly increased in murine and human insulin resistance, and obesity [22]. Pickup et al showed increased levels of IL-6 in persons with more than two features of the metabolic syndrome [23].

### Unifying hypothesis

Therefore, chronic inflammation and increase in CRP level are the common pathways in the metabolic syndrome and infectious agents for promotion of atherosclerotic process. But what is the reason for the association of HSV-1, CMV, H. pylori and Chlamydia pneumoniae and the metabolic syndrome, as we observed in this study. According to a unifying hypothesis, HSV-1, CMV, H. pylori and Chlamydia pneumoniae induce production of proinflammatory cytokines, such as TNF-alpha and IL-6 which are leading to chronic subclinical inflammation, insulin resistance and the metabolic syndrome.

### Limitations

This population-based study showed evidence for the existence of an inflammatory link between HSV-1, CMV, H. pylori and Chlamydia pneumoniae and the occurrence of the insulin resistance dyslipidemic syndrome commonly known as the "metabolic syndrome". However, further studies are needed to confirm our data and elucidate the pathogenic mechanisms underlying metabolic syndrome-infections associations. Our study has some limitations. As with any cross-sectional study design, there is the possibility of unmeasured confounding. A consistent limitation of our seroepidemiologic study, however, is the uncertain correlation between serologic criteria and the presence of endovascular infection. We conducted our study in a large random population and used seropositivity as a marker for infections; however it has the advantage of clinical applicability, but the assessment of infection status based on serology without further clinical or laboratory characterization is subject to diagnostic inaccuracies, especially if seropositivity is common because of the widespread distribution of the incriminated microorganism. But the knowledge of how interaction between metabolic and chronic infectious pathways occurs will be useful in future therapeutic strategies. The effective administration of anti-inflammatory agents, vaccines and antibiotics in the treatment of insulin resistance and atherosclerosis is only the beginning of a promising approach in the management of the metabolic syndrome.

## Conclusion

The metabolic syndrome, which occurs very frequently in the general population, has a significant association with prior infection with Chlamydia pneumoniae, Helicobacter pylori, cytomegalovirus and herpes simplex virus type 1. Hypothesis about participation of infection in pathogenesis of metabolic syndrome should be investigated.

## Abbreviations

National Cholesterol Education Program (NCEP)

Adult Treatment Panel (ATP)

Herpes simplex virus type 1 (HSV-1)

Cytomegalovirus (CMV)

Helicobacter pylori (H. pylori)

Coronary heart disease (CHD)

Cardiovascular disease (CVD)

Ischemic heart disease (IHD)

Enzyme immunounits (EIU)

Odds ratio (OR)

Insulin receptor (IR)

Tumor necrosis factor alpha (TNF-alpha)

## Competing interests

The author(s) declare that they have no competing interests.

## Authors' contributions

IN and KV conceived and designed the study as well as wrote the manuscript; RP assisted in the survey and did statistical analysis; SMJ performed field survey and assisted in immunoassays. ZS performed immunoassays. All authors read and approved the final manuscript.

## References

[B1] Liese AD, Mayer-Davis EJ, Haffner SM (1998). Development of the multiple metabolic syndrome: an epidemiologic perspective. Epidemiol Rev.

[B2] Grundy S (2002). Obesity, metabolic syndrome, and coronary atherosclerosis. Circulation.

[B3] Ford ES, Giles WH, Mokdad  AH (2004). Increasing prevalence of the metabolic syndrome among U.S. adults. Diabetes Care.

[B4] Malik S, Wong ND, Franklin SS, Kamath TV, L'Italien GJ, Pio JR, Williams GR (2004). Impact of the metabolic syndrome on mortality from coronary heart disease, cardiovascular disease, and all causes in United States Adults. Circulation.

[B5] Devaraj S, Rosenson RS, Jialal I (2004). Metabolic syndrome:an appraisal of the proinflamatory and pro-coagulant status. Endocrinol Metabol Clin N Am.

[B6] Siscovick DS, Schwartz SM, Corey L, Grayston JT, Ashley R, Wang SP, Psaty BM, Tracy RP, Kuller LH, Kronmal RA (2000). Chlamydia pneumoniae, herpes simplex virus type 1, and cytomegalovirus and incident myocardial infarction and coronary heart disease in older adults: The Cardiovascular Heart Study. Circulation.

[B7] Aristo V (2003). A look at infectious agents as a possible causative factor in cardiovascular disease. Science.

[B8] Expert Panel on Detection Evaluation and Treatment of High Blood Cholesterol in Adults (2001). Executive summary of the Third Report of the National Cholesterol Education Program (NCEP) Expert Panel on Detection, Evaluation and Treatment of High Blood Cholesterol in Adults (Adult Treatment Panel III). JAMA.

[B9] Xu Q, Schett G, Perschinka H, Mayr M, Egger G, Oberhollenzer F, Willeit J, Kiechl S, Wick G (2000). Serum soluble heat shock protein 60 is elevated in subjects with atherosclerosis in a general population. Circulation.

[B10] Andel M, Tsevegjav A, Roubalova K, Hruba D, louhy P, Kraml P (2003). Infectious and inflammatory factors in the etiology and pathogenesis of atherosclerosis. Vnitr lek.

[B11] Vercellotti GM (2001). Microbs, inflammation and atherosclerosis: will old pathology lessons guide new therapies?. Trans Am Clin Climatol Assoc.

[B12] Willerson JT, Ridker PM (2004). Inflammation as a cardiovascular risk factor. Circulation.

[B13] Ridker PM, Buring JE, Cook NR, Rifai N (2003). C-reactive protein, the metabolic syndrome, and risk of incident cardiovascular events: an 8 year follow up of 14719 initially healthy American women. Circulation.

[B14] Rose R (1999). Atherosclerosis: an inflammatory disease?. N Engl J Med.

[B15] Danesh J, Whincup PI, Walker M, Lennon L, Thomson A, Appleby P, Gallimore JR, Pepys MB (2000). Low grade inflammation and coronary heart disease: prospective study and updated meta-analyses. Br Med J.

[B16] Fernandez-Real JM, Wifredo R (2003). Insulin resistance and chronic cardiovascular inflammatory syndrome. Endocrine Review.

[B17] Fisman EZ, Motro M, Tenenbaum A (2003). Cardiovascular diabetology in the core of a novel interleukins classification: the bad, the good and the aloof. Cardiovasc Diabetol.

[B18] Fisman EZ, Motro M, Tenenbaum A (2003). Regulation of adipocytokines and insulin resistance. Cardiovasc Diabetol.

[B19] Engelman JA, Berg AH, Lewis RY, Lisanti MP, Scherer PE (2000). Tumor necrosis factor-alpha mediated insulin resistance, but not dedifferentiation, is abrogated by MEKl/2 inhibitors in 3T3-L1 adipocytes. Mol Endo.

[B20] Hotamisligil GS, Spiegelman BM (1994). Tumor necrosis factor alpha: a key component of the obesity-diabetes link. Diabetes.

[B21] Castell JV, Gomez-Lechon MJ, David M, Fabra R, Trullenque R, Heinrich PC (1990). Acute phase response of human hepatocytes: regulation of acute-phase protein synthesis by interleukin-6. Hepatology.

[B22] Fasshauer M, Paschke R (2003). Regulation of adipocytokines and insulin resistance. Diabetologia.

[B23] Pickup JC, Mattock MB, Chusney GD, Burt D (1997). NIDDM as a disease of the innate immune system: association of acute-phase reactants and interleukin-6 with metabolic syndrome. Diabetologia.

